# Interruptions of antiretroviral therapy in children and adolescents with HIV infection in clinical practice: a retrospective cohort study in the USA

**DOI:** 10.7448/IAS.19.1.20936

**Published:** 2016-10-27

**Authors:** Natella Rakhmanina, Kam S Lam, Jaclyn Hern, Heather A Young, Alex Walters, Amanda D Castel

**Affiliations:** 1 Division of Infectious Diseases, Children's National Health System, Washington, DC, USA; 2 Department of Pediatrics, School of Medicine and Health Sciences, The George Washington University, Washington, DC, USA; 3 Elizabeth Glaser Pediatric AIDS Foundation, Washington, DC, USA; 4 Rainbow Babies & Children's Hospital, Cleveland, OH, USA; 5 San Francisco Department of Public Health, Substance Use Research Unit, San Francisco, CA, USA; 6 Department of Epidemiology and Biostatistics, Milken Institute School of Public Health, The George Washington University, Washington, DC, USA; 7 School of Medicine, University of South Carolina, Columbia, SC, USA

**Keywords:** HIV, children, treatment interruption, antiretroviral therapy, lamivudine monotherapy

## Abstract

**Introduction:**

Changes in combination antiretroviral therapy (cART) throughout childhood challenge the continuity of paediatric HIV treatment. This study aimed to evaluate the prevalence of treatment interruption (TI), including lamivudine (3TC) monotherapy, and the relationship of TI to virologic and immunologic parameters in HIV-infected paediatric patients.

**Methods:**

Nested within a prospective observational study of a city-wide cohort of HIV-infected persons in the District of Columbia, this sub-study collected retrospective data on antiretroviral therapy, enrolment (endpoint) and historic (lifelong) CD4 counts and HIV RNA viral load (VL) of the paediatric cohort. TI was defined as interruption of cART ≥4 consecutive weeks. Data on TI, including 3TC monotherapy TI (MTI), were collected. Descriptive statistics and univariate testing were used to compare children with TI and MTI to children on continuous treatment (CT).

**Results:**

Thirty-eight (28%) out of 136 enrolled children (median age=12.9 years) experienced TI, with 14 (37%) of those placed on 3TC MTI. Significantly lower endpoint median CD4 counts (598 cells/mm^3^ vs. 815 cells/mm^3^; *p=*0.003) and CD4% (27.5% vs. 33%; *p*=0.006) were observed in the TI cohort as compared to the CT cohort. The median endpoint VL in the overall TI cohort was ~4 times higher than among the CT cohort (1427 copies/mL vs. 5581 copies/mL; *p*<0.0001). After a median TI duration of one year, a majority (*n*=31; 82%) of patients with TI restarted cART, including 100% of those with total TI and 53% of those on MTI, respectively.

**Conclusions:**

In our study, we observed high frequency of the TI in HIV in paediatric HIV clinical practice. All TIs, including 3TC MTI, were associated with significantly lower endpoint median CD4 counts and higher median VLs, as compared to CT in paediatric patients. The high frequency of TI and associated poor outcomes suggest a need for a better strategy in managing the course of the paediatric and adolescent cART.

## Introduction

Combination antiretroviral therapy (cART) has dramatically improved the outcome of paediatric HIV infection. Supported by the evidence from the START trial, the 2015 World Health Organization HIV treatment guidelines recommend a universal start of cART in all HIV-infected individuals including children and adolescents [1,2]. The USA and European guidelines support universal paediatric cART in infants and young children and recommend strong consideration for cART at older age [3,4].

With changes in the child's age and maturity, paediatric cART evolves from the administering liquid and powdered/granulated antiretroviral drugs (ARVs) to swallowing fixed-dose combinations tablets. This transition requires frequent adjustment of ARV dosing based on the child's age and weight and close monitoring for cART tolerability and toxicity. Adherence barriers also continue to evolve from acceptability of paediatric ARVs in infancy to increased peer pressure and disclosure challenges during puberty [5–7]. Moreover, in the areas of highest paediatric HIV epidemic (e.g. sub-Saharan Africa), continuity of paediatric cART is frequently challenged by comorbidities, limited availability of paediatric ARVs and suboptimal paediatric HIV healthcare capacity [8–10].

Together, these challenges create multiple settings for treatment interruptions (TI) of cART from infancy into adolescence. Paediatric and adolescent HIV providers have considered structured TI guided by CD4 count and clinical staging of the disease [11–13]. TI alternatives such as monotherapy with lamivudine (3TC) and lopinavir/ritonavir (LPV/r) have been also considered in children [14–17].

While existing data support the feasibility of structured TI in children, data on the long-term effects of TI in paediatric HIV-infected patients are limited [11,12]. In this study, we evaluated the prevalence and duration of TI, including placement on ARV monotherapy, and compared the virologic and immunologic outcomes between paediatric patients with TI and those on continuous treatment (CT) from a perinatal HIV cohort in Washington, DC, USA.

## Methods

### Data source

This analysis was conducted as part of a larger city-wide longitudinal cohort study of HIV-infected persons receiving care in the District of Columbia (DC), which is aimed to describe clinical outcomes and improve the quality of care for patients in Washington, DC, USA [18]. The paediatric Special Immunology Services (SIS) program at the Children's National Health System (CNHS) is 1 of 13 sites participating in the DC cohort. SIS provides care to ~200 children and adolescents with primarily perinatally acquired HIV. The majority of patients in care are Black (95%) and female (55%).

### Eligibility criteria

HIV-infected children and adolescents (aged 0–24 years) treated at SIS were eligible to participate. Informed consent (parents and participants ≥18 years of age) and assent (7–17 years of age) were required. The study protocol was approved by the Institutional Review Boards (IRBs) at the George Washington University (GWU) and CNHS.

### Study design

Demographic data (age, race and place of birth) and retrospective immunologic (CD4 count and CD4%, lifetime CD4 nadir), virologic (HIV RNA viral load (VL)) and antiretroviral therapy (age at initiations of ARVs and ARVs prescribed) data were collected from the medical records (MR) of patients enrolled into DC cohort during 2011 to 2013. Immunologic and virologic data collected at the time of enrolment through 2013 were used as endpoints across the overall study cohort.

TI was defined as a period of at least *four consecutive weeks of discontinuation of cART*, including placement on monotherapy TI (MTI), approved/prescribed by a clinical provider and documented in the MR. Within the TI cohort, the patients who underwent a TI with no ARVs prescribed were classified as having total TI (TTI). Patients who interrupted cART but were instead prescribed a single ARV 3TC or LPV/r were classified as MTI cohort. A patient with multiple TIs was analyzed as one unit using the data associated with the longest TI period. Patients on cART who did not have TI were classified as the CT cohort. TI data included age at the start and end of TI, reasons for TI, CD4 count and VL closest to the time of starting TI and after the restart of cART, maximal CD4 count prior and after TI and time to reaching maximal CD4 count after restarting cART.

### Data analysis

Frequency distributions and summary statistics, including median and interquartile range (IQR), were used to describe the demographic, clinical and treatment characteristics in general and between those on CT compared to those with TI. Comparisons between groups (CT vs. TI and TTI vs. MTI) were performed using chi-squared and Wilcoxon rank sum tests as appropriate. Among those in the TTI cohort who restarted treatment, maximum CD4 count and VL before and after TI were compared using Wilcoxon sign rank test. Data were analyzed using SAS 9.3 (Cary, NC).

## Results and discussion

### Results

From October 2011 through December 2013, 136 patients were enrolled (Table 1). The majority (97.8%) were perinatally infected. At enrolment, the median CD4 count and CD4% within the overall patient cohort were 726 cells/mm^3^ and 31%, respectively. Approximately half of the study cohort had undetectable or very low VLs with 60 (44%) and 15 (9.4%) patients with HIV RNA VL <48 copies/mL and >48 to <200 copies/mL, respectively (data not shown). Among those with detectable VL, the median HIV RNA VL at enrolment was 3397 copies/mL.

**Table 1:** Characteristics of CT and TI cohorts at enrolment after TI (univariate analysis of associations with demographic and clinical factors)

	Overall cohort (*N*=136)	Continuous treatment (CT) (*N=*98)	Treatment interruption (TI) (*N*=38)	*P*-value (CT and TI)
Demographic parameters				
Age (years)	12.9 (8.3–17.1)^a^	12.4 (8.0–17.1)^a^	14.6 (10.7–16.9)^a^	0.24
Age of ARVs initiation (years)^b^	0.64 (0.3–2.8)^a^	1 (0.3–3.7)^a^	0.5 (0.33–1.5)^a^	0.14
Gender				0.36
Male	63 (46%)	43 (44%)	20 (53%)	
Female	73 (54%)	55 (56%)	18 (47%)	
Race				0.43
Black	117 (86%)	81 (83%)	36 (95%)	
Hispanic/Latino	5 (4%)	4 (4%)	1 (3%)	
Non-Hispanic	9 (7%)	9 (9%)	0 (0%)	
White	5 (4%)	4 (4%)	1 (3%)	
Other/unknown				
Birthplace				0.42
USA	92 (68%)	64 (65%)	28 (74%)	
Africa	17 (13%)	15 (15%)	2 (5%)	
Other^c^	27 (20%)	19 (19%)	8 (21%)	
Immunologic and virologic parameters				
CD4 count (cells/mm^3^)	726 (490–1143)^a^	815 (545–1240)^a^	598 (271–897)^a^	0.003*
CD4%	31 (22–37)^a^	33 (24–37.2)^a^	27.5 (16–33)^a^	0.006*
Nadir CD4 count (cells/mm^3^)	400 (116–608)^a^	429 (134–784)^a^	282 (88–505)^a^	0.04*
HIV RNA VL (copies/mL)	3397 (202–24,928)^a^	1427 (97–9132)^a^	5581 (885–77,484)^a^	<0.0001*

ARVs, antiretroviral drugs; VL, viral load.

a Median (IQR)

b any initial ARVs, including dual and unboosted ARV regimens in older participants

c other included Kazakhstan, Philippines, Trinidad, Ukraine and unknown (not recorded in MR).

* *p*<0.05 are given in bold.

Thirty-eight (28%) participants experienced at least one TI since initiating ARVs. There were six patients with more than one episode of TI lasting at least four weeks. There were no statistically significant differences in demographic characteristics between the TI and CT cohorts (Table 1).

The median CD4 count, CD4% and nadir CD4 count were significantly lower in those who experienced TI as compared to patients in the CT cohort (*p*=0.003, 0.006 and 0.04, respectively; Table 1). There was no difference between the two cohorts in the highest ever CD4 cell count and CD4% after HIV diagnosis (data not shown). The median VL in the TI cohort was almost four times higher than that among children in the CT cohort (1427 vs. 5581 copies/mL; *p*<0.0001).

Among the 38 participants who experienced TI, the median duration of the last cART regimen before TI was 3.97 years and the median age at the time of TI was 10.8 years (Table 2).

**Table 2:** Characteristics of the TI cohort and reasons for TI

TI cohort	*N*=38
Age at TI (years)	10.8 (7.5–15.7)^a^
Duration of last cART regimen prior to TI (years)	3.97 (2.5–7.6)^a^
Maximum CD4 count ever prior to TI (cells/mm^3^)	1509 (1111–1800)^a^
Maximum CD4% ever prior to TI (%)	37 (31–43)^a^
Maximum VL ever prior to TI (copies/mL)	5199 (381–81,758)^a^
CD4 count prior to TI^b^ (cells/mm^3^)	789 (475–1058)^a^
CD4% prior^b^ to TI	27 (22–34)^a^
HIV RNA VL prior^b^ to TI (copies/mL)	2362 (309–65,577)^a^
Reason for TI	
Non-adherence	11 (29%)^c^
ART toxicity	2 (5%)^c^
Viral resistance^d^	4 (11%)^c^
Other^e^	10 (26%)^c^
Unknown^f^	11 (29%)^c^

TI, treatment interruption; VL, viral load; ART, antiretroviral therapy; cART, combination antiretroviral therapy.

a Median (IQR)

b the most recent value before the TI (median=2.3 months (IQR 1.1–8.8 months))

c *N* (%)

d viral resistance to cART with no other treatment options available

e other reasons included difficulties with dose/formulation, high pill burden and ART holiday

f not documented in the MR.

More than half of the patients with TI (*n*=23; 61%) had experienced TTI of cART. Among the remaining 15 (36.8%) children with TI, 14 (93%) patients with known resistance to 3TC were placed on 3TC MTI, and one patient was switched to LPV/ritonavir MTI (data not shown). In the overall TI cohort, the median CD4 count prior to TI was 789 cells/mm^3^ and the median VL was 2362 copies/mL. The most common reason for TI was non-adherence (29%), followed by viral resistance with limited options for alternative cART (11%) and ARV-related toxicity (5%) (Table 2). In 29% of the patients with TI, the reason for stopping cART was not documented. Among 38 patients with TI, 31 (82%) restarted cART after a median TI duration of one year (Table 3).

**Table 3:** Characteristics of patients with TI who restarted cART

Restarted cART	*N*=31^a^
Age at cART restart (years)	12.6 (8.5–16.2)^b^
Duration of TI (years)	0.98 (0.4–2.4)^b^
CD4 count (cells/mm^3^) prior^c^ to restarting cART	493 (270–740)^b^
CD4% prior^c^ to restarting cART	22 (16–30)^b^
HIV RNA VL prior^c^ to restarting cART (copies/mL)	52,002 (3967–129,410)^b^
CD4 count (cells/mm^3^) after^d^ restarting ART	613 (420–1116)^b^
CD4% after^d^ restarting cART (%)	26 (19–35)^b^
HIV RNA VL after^d^ restarting cART (copies/mL)	2510 (129–40,500)^b^
Maximum CD4 count after^d^ restarting cART (cells/mm^3^)	706 (534–1116)^b^
Time to maximum CD4 count after^d^ restarting cART (years)	0.56 (0.2–1.1)^b^

cART, combination antiretroviral therapy; TI, treatment interruption VL, viral load; ART, antiretroviral therapy.

a Patients who restarted cART include 23 patients in TTI cohort and eight patients in MTI cohort

b median (IQR)

c the most recent value before restarting cART while on TI (median=2.3 months; IQR=1.1–8.8 months)

d the most recent value after restarting cART (median=1.8 months; IQR=2 days–4.8 months).

All patients with TTI restarted cART (*n*=23; 100%), while only 8 out of 15 patients (53%) on MTI restarted cART. The highest CD4 count ever reached prior to TI (1509 cells/mm^3^ (IQR 1137–1820)) was significantly higher than the maximal CD4 count ever reached after TI (706 cells/mm^3^ (IQR 531–1140); *p*=0.0005), most likely due to the increased age. TTI and MTI groups did not demonstrate any significant differences in demographic, immunologic or virologic characteristics (data not shown). The only finding in this sub-analysis was the shorter time required by patients in the MTI cohort (two months (IQR 1.4–3.5)) to reach their highest CD4 count after restarting cART as compared to the patients in the TTI cohort (7.9 months (IQR 4.2–22).

### Discussion

Convincing evidence of the detrimental effect of TI on the outcome of HIV disease from adult trials has led to the abandonment of the structured TI as a therapeutic cART approach [19,20]. The TI data from paediatric clinical trials, however, have been more complex [11,12,21]. In resource-limited settings, structured TI of cART has been evaluated as a feasible strategic approach to address limited coverage with paediatric cART [21–23]. CHER trial results have demonstrated that immediate initiation of cART with planned TI resulted in better long-term outcomes compared to the deferred cART [22,23]. Most recently, structured TI of efavirenz-based cART, defined as short-cycle therapy, showed non-inferiority in viral suppression compared to the CT arm in HIV-infected adolescents and young adults [13]. These data advocate for the potential consideration of structured TI in paediatric cART.

The prevalence of TI in our study (28%) was similar to that reported across the observational studies of unstructured TI from developed countries reporting TI in up to 40% of paediatric populations [24–28]. Many of these studies presented data from the early 2000s with limited options for alternative paediatric cART and higher ARV-associated toxicity. Our retrospective cohort included patients up to 17 years of age, some with a history of dual and unboosted cART, which might have resulted in limited choices for replacement regimens. Similar to findings from adult and paediatric studies from the USA and Europe, the most common reason for TI in our study was non-adherence to cART [24,26–29].

Several studies have suggested higher immunologic resilience and rebound following prolonged TI in children due to the inherent differences in immune system maturation compared to adults [11,12,21,23]. Despite the apparent tolerability of TI, concern for the delayed formation of drug resistance and long-term immunologic effect in children remained [11]. In a recent analysis of the three-year follow-up of the ARROW trial, every month of TI was associated with a 2% (1–3%, *p*=0.001) decrease in CD4% among children and the history of any TI was associated with a trend to increased mortality (hazard ratio: 2.6 (95% CI 0.7–10.4)) [21]. In the CHER study, the majority (80%) of children in the TI arm reached immunologic and clinical criteria requiring restarting cART after 4.8 years [23]. Consistent with these studies, in our paediatric cohort, the endpoint immunologic parameters in the patients with TI were significantly lower as compared to those on CT.

Structured TI has been linked to *holding* or *bridging* regimen using 3TC or emtricitabine (FTC) monotherapy in the presence of the M184V HIV resistance mutation to reduce viral fitness and prevent formation of additional resistance mutations [14,15]. IMPAACT P1094 study compared the immunologic outcome of continuing failing cART versus switching to 3TC/FTC MTI in perinatally infected non-adherent adolescents [14]. Participants (*n*=17) randomized to 3TC MTI were more likely than those maintained on failing cART (*n*=16) to sustain a ≥30% decline in CD4 count [14]. Recent observational data from 71 South African children on 3TC MTI as an alternative to failing cART reported that a majority of children (67.5%) on 3TC MTI had a >25% decline in CD4 count; however, only 23% were switched to second- or third-line cART [15]. In our study, all patients in the TTI group were restarted on cART, whereas less than half (46%) in the 3TC MTI cohort restarted cART. We did not observe significant immunologic or virologic differences between those on TTI and on 3TC MTI, although a shorter time was required by patients on 3TC holding regimen to reach their highest CD4 count after restarting cART. The single site and small sample size limits our ability to generalize these findings.

## Conclusions

Due to the developmental changes throughout childhood and adolescence, TI represents an ongoing challenge to the continuum of paediatric cART. Prolonged TI can have potentially detrimental effects on the long-term outcomes in HIV-infected children. In our study, significantly lower median CD4 count and higher median VL were observed in children with a history of TI, including patients on 3TC MTI, as compared to those on continued cART. A sub-group analysis suggests a trend for a shorter time for 3TC MTI cohort to reach their highest CD4 count after restarting cART, which warrants further evaluation. Finally, the high frequency of TIs reported in our and other paediatric studies suggests a need for a better strategy in maintaining the continuity of the paediatric and adolescent cART.
